# Inhaled budesonide for adults with mild-to-moderate asthma: a randomized placebo-controlled, double-blind clinical trial

**DOI:** 10.1590/S1516-31802001000500004

**Published:** 2001-09-01

**Authors:** Ana Luisa Godoy Fernandes, Sonia Maria Faresin, Maria Marta Amorim, Carlos Cézar Fritscher, Carlos Alberto de Castro Pereira, José Roberto Jardim

**Keywords:** Asthma, Inhaled corticosteroid, Budesonide, Asma, Corticóide inalatório, Budesonida

## Abstract

**CONTEXT:**

Budesonide is an inhaled corticosteroid with high topical potency and low systemic activity recommended in the treatment of chronic asthma.

**OBJECTIVE:**

This study was conducted to determine the efficacy and safety of inhaled budesonide via a breath-activated, multi-dose, dry-powder inhaler.

**TYPE OF STUDY:**

Multicenter randomized parallel-group, placebo-controlled, double-blind, clinical trial.

**SETTING:**

Multicenter study in the university units.

**PARTICIPANTS:**

Adult patients with mild-to-moderate asthma that was not controlled using bronchodilator therapy alone.

**PROCEDURES:**

Comparison of budesonide 400 μg administered twice daily via a breath-activated, multi-dose, dry-powder inhaler with placebo, in 43 adult patients (aged 15 to 78 years) with mild-to-moderate asthma (FEV1 71% of predicted normal) that was not controlled using bronchodilator therapy alone.

**MAIN MEASUREMENTS:**

Efficacy was assessed by pulmonary function tests and asthma symptom control (as perceived by the patients) and the use of rescue medication.

**RESULTS:**

Budesonide 400 μg (bid) was significantly more effective than placebo in improving morning peak expiratory flow (mean difference: 67.9 l/min; P < 0.005) and FEV1 (mean difference: 0.60 l; P < 0.005) over the 8-week treatment period. Onset of action, assessed by morning peak expiratory flow, occurred within the first two weeks of treatment.

**CONCLUSIONS:**

Budesonide via a breath-activated, multi-dose, dry-powder inhaler results in a rapid onset of asthma control, which is maintained over time and is well tolerated in adults with mild-to-moderate asthma.

## RESUMO

**CONTEXTO:**

A budesonida é um corticóide inalatório de alta potência tópica e baixa atividade sistêmica recomendado no tratamento da asma crônica.

**OBJETIVO:**

Determinar a eficácia e tolerabilidade da budesonida via um inalador em pó com atividade inspiratória e multidose, comparada com placebo, em pacientes adultos portadores de asma leve ou moderada não-controlados com terapia broncodilatadora.

**TIPO DE ESTUDO:**

Estudo randomizado com dois grupos paralelos, duplo-cego, placebo controlado.

**LOCAL:**

Estudo multicêntrico em centros universitários.

**PARTICIPANTES:**

43 pacientes adultos (idade entre 15 e 78 anos), portadores de asma leve ou moderada (VEF1 71% do normal previsto) não-controlados com terapia broncodilatadora.

**PROCEDIMENTOS:**

Para comparar, com placebo, a eficácia e tolerabilidade de 400 μg de budesonida, administradas duas vezes ao dia, via turbuhaler.

**VARIÁVEIS ESTUDADAS:**

A eficácia foi determinada através de testes de função pulmonar, controle dos sintomas de asma e uso de medicação de resgate.

**RESULTADOS:**

400 μg de budesonida duas vezes ao dia foram significantemente mais efetivas que placebo em relação ao aumento do peak expiratory flow matutino (67,9 l/min. p < 0,005) e VEF1 (0,60 l/min., p < 0,05) nas oito semanas de tratamento.

**CONCLUSÃO:**

O tratamento com budesonida via um inalador em pó com atividade inspiratória e multidose, em pacientes portadores de asma leve e moderada, é bem tolerado e resulta em rápido e duradouro controle da asma.

## INTRODUCTION

Asthma is one of the most frequent respiratory diseases, in patients of any age, gender and/or race. Prevalence is around 10% to 15% in children all over the world.^1–3^

The asthma treatment paradigm has changed over the past 10 years, with preventive treatment of underlying airway inflammation using inhaled corticosteroids replacing the previous emphasis on symptom relief with short acting beta agonists.

Some meta-analyses comparing the newer inhaled corticosteroids budesonide and fluticasone propionate to beclomethasone dipropionate have shown evidence of limitations in improvement in pulmonary function and other variables. Fluticasone propionate at half dose was more effective than budesonide and as effective as beclomethasone dipropionate in improving peak expiratory flow rate.^4^ However, the limited availability of data with similar criteria for symptom monitoring has made it difficult to compare studies using fluticasone propionate, budesonide and beclomethasone dipropionate.^5^

Randomized placebo-controlled, double-blind, clinical trials with adequate methodology may be a better way to demonstrate results than studies with a large number of patients but less uniform data collection.

Budesonide is an inhaled corticosteroid with high topical potency and low systemic activity. Budesonide via a breath-activated, multi-dose, dry-powder inhaler is recommended in the treatment of chronic asthma.^6^

The clinical efficacy of an inhaled drug is greatly influenced by the device used to deliver the drug into the lungs. Factors such as pulmonary deposition and compliance with treatment may influence the results of a comparative study on the efficacy of two drugs. Pulmonary deposition of budesonide in healthy volunteers has been shown to be 32% of the nominal dose when using a breath-activated, multi-dose, dry-powder inhaler, and between 15 - 18% when using pressurized metered dose inhalers.^7^

Broad clinical experience has demonstrated the efficacy and tolerability of budesonide in the treatment of asthma in adults and children.^8–11^

There is evidence that inhaled corticosteroid in the treatment of moderate asthma may reduce the need for oral corticosteroid treatment. However, some questions remain regarding the real benefits of this therapy for patients with mild asthma.^12^

Some clinicians are skeptical about transferring meta-analysis results to clinical practice. Thus, the use of budesonide for mild-to-moderate stable asthmatics must be rigorously evaluated in randomized controlled clinical trials.

The objective of this study was to assess the efficacy and tolerability of budesonide 400 μg (bid) in asthmatic patients for 8 weeks, compared with a placebo, both administered via a breath-activated, multi-dose, dry-powder inhaler. The primary efficacy variables were: change from baseline in forced expiratory volume in the first second (FEV1) and peak expiratory flow.

## METHODS

The procedures that follow were in accordance with the ethical standards of the committee responsible for human experimentation and with the Helsinki Declaration of 1975, as revised in 1983. All three Institutions had approval from Independent Ethics Committees for conducting the study.

### General design of the study

This was a randomized, double-blind, placebo-controlled, multicenter study, performed at two Universitary centers and one reference center for asthmatic patients (Hospital São Paulo, Universidade Federal de São Paulo/Escola Paulista de Medicina, São Paulo, n = 22; Respiratory Division, Hospital do Servidor Público Estadual, São Paulo, n = 12; Pneumology Division, Pontifícia Universidade Católica, Rio Grande do Sul, n = 9) with parallel groups, from June 1997 to January 1998, with eight weeks of follow-up. All the studied drug was stored in multidose dry powder inhalers with same appearance and 200 doses/device. The drug was prepared and labeled by the pharmaceutical company. 60 devices were numbered, and divided between budesonide or placebo. The randomization was performed before the delivery to the centers. The blind was kept on during preparation and the trial, and the seal was opened before the analyses.

### Participants

*Inclusion criteria*: Patients with mild-to-moderate asthma, according to the international guidelines for diagnosis and management of asthma, were included. They had pre-bronchodilator FEV1 of between 40% and 90% of the predicted normal value at visit one (reference values from the Brazilian Council of Spirometry^15^) and an increase of 12% and 200 ml in FEV1 after 500 μg of terbutaline sulfate (Bricanyl TurbohalerÒ). After receiving instructions, the patients had to be able to use the Turbohaler according to the product utilization techniques, as well as fill in the diary correctly. During the run-in period the patient were to use the rescue medication at least 6 times in 7 days (but never exceeding 3 inhalations/day), or for night awakenings due to asthma at least 2 times during the 14 days.

*Exclusion criteria:* Pregnant or breast-feeding women, and individuals with other chronic pulmonary diseases, clinically relevant respiratory infections, or participation in other drug investigation studies during the 4 weeks prior to visit 1, were excluded.

### Procedures

Patients stopped their usual glicocorticosteroid inhaler therapy 2 weeks prior to inclusion in the run-in period and they were asked to complete a diary card every day, in the morning and evening, with their asthma symptoms, need for rescue medication, and peak expiratory flow. The use of beta-2 agonist was allowed for symptom relief, and was to be recorded in the diary cards.

After the run-in period, patients who had asthma symptoms according to GINA^10^ were randomized into one of the two treatment groups, taking either two daily doses of budesonide 400 μg (Inhaled corticosteroid: Pulmicort Turbohaler 200 μg/dose, Astra Draco AB, Sweden) or placebo (Placebo Turbohaler, Astra Draco AB, Sweden). Patients were also requested to rinse their mouths after the use of the inhaled medication.

The patients were instructed to contact the investigator every time they had to double the use of rescue medication, if the symptom score increased and/or if they had a reduction of 30% or more in their peak flow measurement compared with baseline. Every time this happened it was considered that the patient was having an asthma exacerbation, and a course of prednisolone 40 mg a day for 5 days was prescribed. Three consecutive courses of oral corticosteroids or hospitalization due to asthma symptoms were considered to be treatment failure, and such patients were withdrawn from the study.

Patients were asked to come to the center every 14 days and were requested to avoid the use of rescue medication during the 6 hours prior to spirometry and clinical evaluations.

### Main outcome measurements

The primary efficacy variables were: change from baseline in forced expiratory volume in the first second (FEV1) and peak expiratory flow. Secondary variables were: presence of asthma symptoms, use of rescue medication and number of exacerbations during the study period. Peak expiratory flow, symptoms, and bronchodilator use over the 14 days before the visit were obtained from diary cards.

*FEV1 and peak expiratory flow.* During each visit, besides clinical evaluation, spirometry was performed according to the Brazilian Consensus of Spirometry, and the following parameters were measured: FEV1 (forced expiratory volume in one second) and FVC (forced vital capacity), before and after the use of bronchodilator (terbutaline sulfate 0.5 mg).^13,14^ During all visits, patients were asked about any adverse event, whether or not it had any relationship to the study treatment.

*Investigator's opinion*. At the end of the study, the investigator's opinion about efficacy and tolerability of the drug was obtained in comparison with the run-in period: Excellent - complete control of symptoms; Good - few symptoms; Medium - reduction but persistence of symptoms; Insufficient - same symptoms.

*Asthma symptoms.* Every morning and evening, the patient performed an evaluation of his/her asthma symptoms over the preceding day or night period. This evaluation included an overall assessment of the following symptoms: shortness of breath, chest discomfort, wheezing and cough. The following scale was used to record these combined symptoms: Point scale for nocturnal symptoms: 0 = no night awakening due to asthma; 1 = one night awakening due to asthma; 2 = more than one night awakening due to asthma; 3 = could not sleep due to asthma; Point scale for daytime symptoms: 0 = no symptoms at all, unrestricted activity; 1 = symptoms caused little or no discomfort, unrestricted activity; 2 = symptoms caused some discomfort, at times limiting strenuous activity; 3 = symptoms caused moderate discomfort, limited routine activity.

The number of inhalations taken during the day and night were recorded in the patient diary card. Patients were also requested to record the peak expiratory flow in l/min daily, in the morning and evening, before taking the study medication and, preferably, at least 6 hours after rescue medication use. On each occasion they were asked to measure peak expiratory flow three times, while standing up, and record the highest value in the diary.

### Statistical methods

*Calculation of the sample size.* It was estimated that up to the 60^th^ patient should be included (there were 60 medications prepared for randomization).

*Statistical analysis.* Student's t test was utilized for comparisons of the numerical variables and the chi-squared test for comparisons of category variables between budesonide and placebo. Friedman's test was applied to observe variables in each individual and the Mann-Whitney test for comparison of variables in individuals between groups (budesonide or placebo) at each visit. For statistical calculations of the score values obtained from the diaries, the reference value was the average of the values in the run-in period for each patient. For all tests, a level of 0.05 or 5% was established for rejection of the null hypothesis.

## RESULTS

### Protocol deviations

Of the 43 adult patients selected for the run-in period, 23 were randomized to the budesonide group and 20 to the placebo group. Forty-one patients completed the 8 week randomized period of the study. Two patients, one from each group, were excluded from the study because the occurrence of severe acute of asthma exacerbation

### Baseline characteristics

No significant differences were observed among subject characteristics at the time of inclusion in the protocol (Table 1). Table 2 shows the variables studied and the changes from baseline that were statistically significant have been marked with an asterisk.

**Table 1:** Subjects characteristics before randomization (visit 0)

*Chracteristics*	*Groups*		*P*
	*Budesonide 800 μg* *n = 23*	*Placebo* *n = 20*	
*Age, years (SD).*	*31.7 (15.5)*	*36.2 (13.2)*	*n.s.*
*Male (%).*	*8 (34.8)*	*4 (20)*	*n.s.*
*Female (%).*	*15 (65)*	*16 (8)*	*n.s.*
*FEV1 pre-bronchodilator liters (%).*	*2.3 (71.5)*	*2.2 (77.5)*	*n.s.*
*FEV1 post-bronchodilator liters (%).*	*2.7 (83.3)*	*2.6 (77.50)*	*n.s.*

***.Mann-Whitney test: not significant (n.s.).***

**Table 2:** Forced expiratory volume pre- and post-bronchodilator, morning peak flow, day and night symptom scores, and beta agonist usage over the trial

	*Grupos*		Comparison between groups**
	*budesonide*	*placebo*	
***FEV1 pre bd***			
*week. −2*	*2.3 (SD 0.7)*	*2.1 (SD 0.8)*	*n.s.*
*week. 0*	*2.3 (SD 0.8)*	*2.2 (SD 0.7)*	*n.s.*
*week. 2*	*2.7 (SD 0.6)*	*2.0 (SD 0.9)*	*p < 0.05*
*week. 4*	*2.8 (SD 0.5)*	*2.1 (SD 0.9)*	*p < 0.05*
*week6*	*-*	*-*	*-*
*week8*	*2.7 (SD 0.7)*	*2.1 (SD 0.7)*	*p < 0.05*
p*	*p < 0.001*	*n.s.*	
***FEV1 post bd***			
*week. −2*	*2.8 (SD 0.6)*	*2.7 (SD 0.7)*	*n.s.*
*week. 0*	*2.7 (SD 0.5)*	*2.6 (SD 0.5)*	*n.s.*
*week. 2*	*3.0 (SD 0.7)*	*2.4 (SD 0.8)*	*p < 0.05*
*week. 4*	*3.0 (SD 0.6)*	*2.5 (SD 0.6)*	*n.s.*
*week6*	*-*	*-*	*-*
*week8*	*3.0 (SD 0.7)*	*2.5 (SD 0.7)*	*n.s.*
p*	*n.s.*	*n.s.*	
***morning peak expiratory flow***			
*week.0*	*329.8 (SD 99.6)*	*301.1 (SD 71.5)*	*n.s.*
*week.2*	*348.2 (SD 117.1)*	*294.6 (SD 73.6)*	*p < 0.05*
*week.4*	*375.3 (SD 112.4)*	*313.8 (SD 64.0)*	*p < 0.05*
*week6*	*385.6 (SD 118.3)*	*317.7 (SD 75.1)*	*p < 0.05*
*week8*	*387.1 (SD 120.8)*	*319.2 (SD 67.9)*	*p < 0.05*
p*	*p < 0.001*	*n.s.*	
***day symptoms***	*(Min-Max)*	*(Min-Max)*	
*week.0*	*0.9 (0.07- 2.3)*	*0.6 (0.07 - 1.1)*	*n.s.*
*week.2*	*0.5 (0.0 - 2.2)*	*0.8 (0.0 - 3.0)*	*n.s.*
*week.4*	*0.4 (0.0 -1.7)*	*0.7 (0.0 - 1.4)*	*n.s.*
*week6*	*0.5 (0.0 - 2.1)*	*0.6 (0.0 - 1.5)*	*n.s.*
*week8*	*0.3 (0.0 - 1.7)*	*0.5 (0.08 - 1.1)*	*n.s.*
p*	*p*	*< 0.01 n.s.*	
***night symptoms***	*(Min-Max)*	*(Min-Max)*	
*week. 0*	*0.9 (0.1 - 2.3)*	*0.7 (0.07 - 1.7)*	*n.s.*
*week. 2*	*0.5 (0.0 - 2.2)*	*0.7 (0.0 - 1.5)*	*n.s.*
*week. 4*	*0.4 (0.0 - 1.6)*	*0.6 (0.0 - 1.4)*	*n.s.*
*week6*	*0.4 (0.0 - 1.6)*	*0.6 (0.0 - 1.5)*	*n.s.*
*week8*	*0.4 (0.0 − 1.7)*	*0.5 (0.08 - 1.1)*	*n.s.*
p*	*p < 0.01*	*n.s.*	
***beta 2 use: day***			
*week. 0*	*1.0 (SD 0.7)*	*0.8 (SD 0.5)*	*n.s.*
*week. 2*	*0.7 (SD 0.7)*	*0.9 (SD 0.7)*	*n.s.*
*week. 4*	*0.5 (SD 0.5)*	*0.8 (SD 0.6)*	*n.s.*
*week6*	*0.4 (SD 0.5)*	*0.7 (SD 0.7)*	*n.s.*
*week8*	*0.4 (SD 0.5)*	*0.6 (SD 0.5)*	*n.s.*
p*	*p < 0.01*	*n.s.*	
***beta 2 use: night***			
*week. 0*	*1.1 (SD 0.7)*	*1.2 (SD 0.5)*	*n.s.*
*week. 2*	*0.9 (SD 0.7)*	*1.3 (SD 0.7)*	*n.s.*
*week. 4*	*0.9 (SD 0.5)*	*1.1 (SD 0.6)*	*n.s.*
*week6*	*0.8 (SD 0.5)*	*1.1 (SD 0.7)*	*n.s.*
*week8*	*0.8 (SD 0.5)*	*1.0 (SD 0.5)*	*n.s.*
p*	*p = 0.06*	*n.s.*	

* ***comparison over time - Friedman test.***

** ***comparison between groups - Mann Whitney test. bd = bronchodila***

### Main outcomes measurements

The pre-bronchodilator FEV1 values showed a significant increase from 2.3 l to 2.8 l in the budesonide group (P < 0.001) after the 2nd week of treatment, in comparison with the placebo group (P < 0.05) (Figure 2).

**Figure 1:**
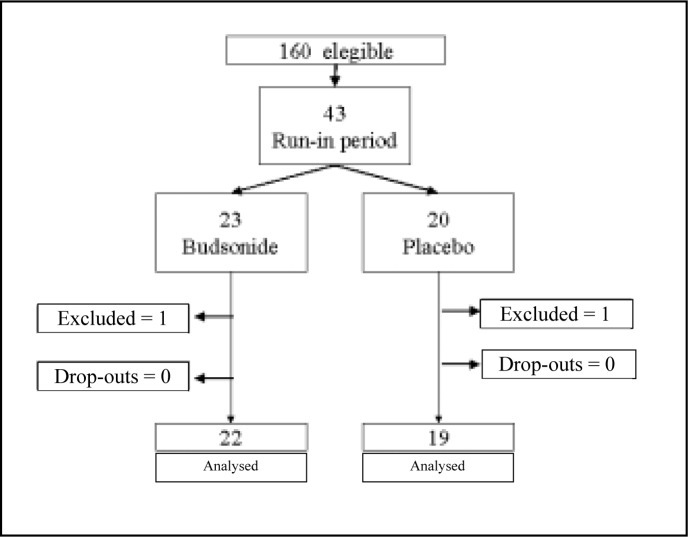
Study profile.

**Figure 2:**
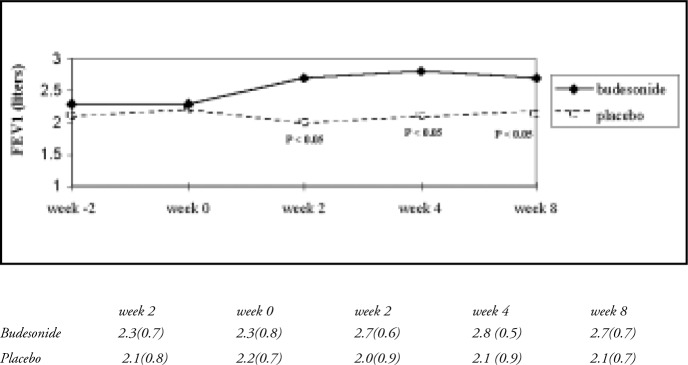
Changes in FEV1 over 8 weeks, using budesonide (solid line) and placebo (dotted line).

Morning peak expiratory flow values were significantly higher in the budesonide group (P < 0.001) after 2 weeks of treatment. The symptom score (diurnal and nocturnal) showed significant reduction in the budesonide group, as well as the use of beta-2 agonist for symptom relief, after 2 weeks of treatment (Table 2).

Three patients in the budesonide group and 5 in the placebo group presented asthma exacerbation and took a course of prednisone 40 mg daily for 5 days during the trial. Although there was a higher number of exacerbations in the placebo group, this difference did not reach statistically significant values (P = 0.27). There was no difference in the occurrence of adverse events between the two groups.

Table 3 presents the efficacy of the treatment according to the investigator's opinion.

**Table 3:** Efficacy of treatment according the investigator opinion.

*Scores*	*group* *budesonide*		*group* *placebo*		*P*
	*n*	*%*	*n*	*%*	
*Excelent*	*9*	*40.9*	*3*	*16.7*	*n.s.*
*Good*	*5*	*22.7*	*5*	*27.8*	
*Medium*	*4*	*18.2*	*4*	*22.2*	
*Insuficient*	*4*	*18.2*	*6*	*33.3*	
***Total***	***22***	***100.0***	***18***	***100.0***	

***Qui-square test: n.s.= not significant.***

***Investigator opinion compared with run-in period***

***Excelent: complete control of symptoms, Good: minimum symptoms, Medium: reduction but persistence of symptoms, Insufficent: same expression of symptoms.***

## DISCUSSION

Asthma is a chronic disease with variable clinical expression and airway inflammation underlies all levels of asthma severity. To achieve good clinical control, patient monitoring through assessment of symptoms and clinical signs is needed.

This study showed that treatment with budesonide via Turbohaler of adults with mild-to-moderate asthma has a rapid onset of disease control, which is maintained for more than eight weeks and is well tolerated. It was the first time that second-generation inhaled glucocorticosteroids had been tried in our country. The study was especially designed to test efficacy and safety of budesonide in asthmatics

Patients had had their asthma diagnosed at least one year prior to entering the study, with persistent clinical symptoms and expression of mild-to-moderate asthma according to GINA.^10^ To prove its efficacy, budesonide 800 μg/day (bid) was used and the follow-up included the monitoring of bronchial obstruction, asthma symptoms and rescue medication use, as well as the occurrence of asthma exacerbation during the trial.

Several studies have shown the efficacy of inhaled long-acting beta-2 agonists for mild-to-moderate asthma, leading to a reduction of diurnal and nocturnal symptoms and an improvement in pulmonary function. A double-blind study with a combination of formoterol to budesonide in high and low doses (FACET study) showed good clinical control, but the number of severe exacerbations was even lower in patients with higher doses of budesonide. Increasing the maintenance dose of inhaled corticosteroid might be a more appropriate initial treatment for patients with unstable asthma. However, decisions about appropriate therapy will depend upon the patient's medical history, symptoms, clinical characteristics and previous treatment choices.^15,16^

The clinical protocol was designed such that patients with symptomatic asthma had the disease confirmed by the presence of daytime and evening symptoms of mild-to-moderate asthma during the run-in period of two weeks. The improvement in asthma control was linked to the budesonide treatment twice daily. Our patients presented a significant reduction in the symptom score and use of rescue medication associated with budesonide 800 μg/day (Table 2). If we consider that asthma is an inflammatory disease in which the clinical manifestations are a consequence of inflammatory mediators, the use of beta-2 agonist for symptom relief is a good parameter for evaluation of the inflammatory activity of the disease. According to Busse et al,^17^ the reduction in asthma attacks with the regular use of budesonide means an improvement in the inflammatory process of the bronchial mucosa.

The patients’ pulmonary functions and clinical conditions did not differ before randomization (visit 2) (Table 1). Thus, both groups started the study under the same conditions, which guaranteed that observed results reflected the given therapy.

Several studies in the medical literature have shown the efficacy of inhaled corticosteroids in the control of moderate asthma,^18^ leading to a reduction in diurnal and nocturnal symptoms and an improvement in pulmonary function, as shown by this study.

At the time this study was performed, the long-lasting beta-2 was not available in Brazil, and the second generation of inhaled corticosteroids such as budesonide had just been launched in the market. This special situation in comparison with other countries gave us the opportunity to make comparisons between the drug and placebo, even in patients with additional asthma symptoms. With the introduction of all kinds of second-generation inhaled corticosteroids associated with long-lasting beta-2 agonist, it has become less frequent to follow patients in a study to obtain results that reflect only the use of inhaled corticosteroids.

Busse et al,^19^ in another multicenter study (473 subjects; baseline FEV1 = 63 to 66% of predicted values), showed that the efficacy of budesonide is not dose-dependent. They studied patients with total daily doses of 200, 400, 800 or 1600 μg and observed that all doses were better than placebo and there was no difference between 400 and 800 μg. The improvement in FEV1 and peak expiratory flow over 12 weeks of study was also statistically significant.

The morning and evening peak expiratory flow measurements (Table 2) showed a statistically significant increase, tending to be maintained until the end of the study, providing a larger bronchial caliber in patients using budesonide. The average peak expiratory flow improvement in the budesonide group was 60 l/min. In the multicenter FACET study with 852 asthmatic subjects, the group that used high doses of budesonide without beta-2 agonist did not experience great improvement, but patients using a long-acting beta-2 agonist in combination with low or high doses of budesonide showed a 40 l/min increase in morning peak expiratory flow. In comparison, we obtained this result without the use of a bronchodilator.

However, in contrast to our study, FACET did not have a placebo group. Even with a small number of patients in comparison with the large multicenter studies, our results showed that budesonide 800 μg was significantly more effective than placebo in improving morning peak expiratory flow (a mean difference from placebo of 67.9 l/min; P < 0.005).

FEV1 values improved significantly (400 ml; Table 2) in patients who used budesonide (a mean difference from placebo of 0.60 l; P < 0.005) over the 8-week treatment period. This gain represents the obtaining of good control over the symptoms and a guarantee of minimizing the remodeling process associated with the chronic respiratory inflammation present in asthmatic airways.

Enright et al.^20^ considered the gain in FEV1 the best parameter for the follow-up of asthmatic patients, since it represents the improvement in pulmonary function during one observational period and the best condition that a patient can attain after maximum therapy on the evaluation day. This has a better clinical correlation with good asthma control than bronchial responsiveness tests for histamine or metacholine.

The frequency of asthma exacerbations did not differ between the two groups of patients. There were 5 occurrences in the placebo group and 3 in the budesonide group.

A recent review of the FACET study has concluded that exacerbations may be predominantly associated with a change in symptom score and in peak expiratory flow, but the pattern (intensity of variation) was not affected by the dose of inhaled corticosteroid or whether the patient was taking formoterol.^21^

The side effects were comparable for the budesonide and placebo groups, and the tolerability was considered good for both groups.

The efficacy interpreted by the investigator did not differ statistically between the groups (Table 3). However, there was a predominance of excellent scores in the budesonide group (40%), in comparison with the placebo group (16.7%).

## CONCLUSION

Budesonide is an effective drug for obtaining good control of patients’ asthma, minimizing the symptom score and reducing the use of relief medication. There was an improvement in pulmonary function, with a significant increase in the values of FEV1 and peak expiratory flow.
